# A Guided Wave Transducer with Sprayed Magnetostrictive Powder Coating for Monitoring of Aluminum Conductor Steel-Reinforced Cables

**DOI:** 10.3390/s19071550

**Published:** 2019-03-30

**Authors:** Fuzai Lv, Pengfei Zhang, Zhifeng Tang, Yonggang Yue, Keji Yang

**Affiliations:** 1 State Key Laboratory of Fluid Power and Mechatronic Systems, School of Mechanical Engineering, Zhejiang University, 38 Zheda Road, Hangzhou 310027, China; lfzlfz@zju.edu.cn (F.L.); zhangpengfei@zju.edu.cn (P.Z.); yangkj@zju.edu.cn (K.Y.); 2 Institute of Advanced Digital Technologies and Instrumentation, College of Biomedical Engineering & Instrument Science, Zhejiang University, 38 Zheda Road, Hangzhou 310027, China; 3 Inner Mongolia EHV Power Supply Bureau, Hohhot 010080, China; hvyue@163.com

**Keywords:** guided wave detection, magnetostriction, multi-wire cable, sprayed magnetostrictive powder coating transducer

## Abstract

Aluminum conductor steel-reinforced (ACSR) cables are typically used in overhead transmission lines, requiring stringent non-destructive testing owing to the severe conditions they face. Ultrasonic guided wave inspection provides promising online monitoring of the wire breakage of cables with the advantages of high sensitivity, long-range inspection, and full cross-sectional coverage. It is a very popular method to generate and receive guided waves using magnetostrictive and piezoelectric transducers. However, uniformly coupling the acoustic energy excited by transducers into multi-wire structures is always a challenge in the field application of guided waves. Long-term field application of piezoelectric transducers is limited due to the small coupling surface area, localized excitation, and couplant required. Conventional magnetostrictive transducers for steel strand inspection are based on the magnetostrictive effect of the material itself. Two factors affect the transducing performance of the transducers on ACSR cables. On one hand, there is a non-magnetostrictive effect in aluminum wires. On the other hand, the magnetostriction of the innermost steel wires is too weak to generate guided waves. The bias magnetic field is attenuated by the outer layers of aluminum wires. In this paper, an alternative sprayed magnetostrictive powder coating (SMPC) transducer was developed for guided wave generation and detection in ACSR cables. The Fe_83_Ga_17_ alloy powder with large magnetostriction was sprayed uniformly on the surfaces of certain sections of the outermost aluminum wires where the transducer would be installed. Experimental investigations were carried out to generate and receive the most commonly used L(0,1) guided waves for wire breakage detection at frequencies of 50 and 100 kHz. The results demonstrate that the discernable reflected waves of the cable end and an artificial defect of three-wire breakage (5.5% reduction in the cable’s cross-sectional area) were received by the transducer with SMPC, which was impossible for the transducer without SMPC. This method makes long-term and online monitoring of ACSR cables feasible due to the high coupling efficiency and good structural surface adaptability.

## 1. Introduction

Multi-wire cables are widely used in a number of engineering applications to meet various demands, such as load carrying in elevators, lifting machinery, and cable-stayed and suspension bridges; post-tensioning in civil structures; and as overhead transmission lines (OVTLs) in power grids. These cables span long distances and are subject to large tensile stresses. Aluminum conductor steel-reinforced (ACSR) cables are commonly used in OVTLs. They are influenced by environmental factors such as icing [1], wind-induced vibrations [2], and lightning strikes [3]. Some cases [4,5,6] of ACSR cable structural failures caused by these factors have been reported in recent years.

The safety of the structures depends on the structural integrity of these cables, which have stringent non-destructive testing (NDT) and online structural health monitoring (SHM) requirements owing to the severe conditions they face. There are many NDT methods, such as visual inspection [7], radiography [8], computed tomography [9], acoustic emission monitoring [10,11], ultrasound [12,13], magnetic flux leakage [14], and eddy current [15]. However, many of these methods are limited in the application of multi-wire cables. For instance, conventional ultrasonic excitation methods using bulk waves are impractical for inspecting large or long structural components like cables, since they can only cover a small section of the structure at a time. Recently, ultrasonic guided wave (UGW)-based techniques have been utilized for the inspection of various structures based on their properties of high sensitivity, long-range inspection, and full cross-sectional coverage [16,17,18]. Many studies related to guided wave dispersion, propagation characterization, and defect detection in multi-wire strands and ropes [19,20,21,22,23,24] have been reported.

Many techniques have emerged and have been used to take precautions against ACSR cable failure. Unmanned aerial vehicle (UAV) based visual inspection has achieved some good results in cable surface condition inspection in place of human eyes from the ground [25]. Autonomous robots for overhead transmission line inspection can automatically move along the cable and record inspection information [26]. Salazar et al. used two wireless piezoelectric transducers to excite and receive guided waves in an 0.9 m long ACSR cable with artificial defects [27]. Baltazar et al. found a mode conversion phenomenon in pitch-catch guided wave detection experiments using two piezoelectric transducers pasted on the end surfaces of the ACSR cable [28]. The detection range of an ACSR cable is limited by multi-wire using guided waves. Legg proposed a dispersion compensation method to increase the detection range [29]. To the best of the authors’ knowledge, the guided wave transducers used in these ACSR cable detection cases were piezoelectric and required couplant.

Piezoelectric [20,30] and magnetostrictive [31,32] transducers have been commonly used to generate and receive guided waves, including in the aforementioned studies. However, for practical implementation, both types of transducers are not capable of meeting the requirements of high coupling efficiency, uniform acoustic field distribution in the cross-section of the cable, long propagation distance, and stable installation. The main limitations are the multilayer structure composed of different materials and the individual wires with small diameters.

Magnetostrictive patch transducers (MPTs) are widely used to generate and measure torsional mode-guided waves in pipes and tubes [32]. The pre-magnetized magnetostrictive patch is pasted to the circumference of the pipe surface as part of the transducer [33]. Since the irregular surface of steel strands does not allow use of the magnetostrictive patch, the magnetostrictive effect of the steel wires themselves was applied to generate and receive guided waves. Unfortunately, the application of these two types of conventional magnetostrictive transducers is limited in ACSR cable inspection using guided waves by some obvious factors. There is a non-magnetostrictive effect in aluminum wires, and the magnetostriction of the innermost steel wires is too weak to generate guided waves due to the layers of aluminum wires. It is also difficult to paste the magnetostrictive patch on the surface of aluminum wire because the patch is fragile and unable to bend.

On the other hand, the performance of the piezoelectric transducers is not good because of the localized excitation and low coupling efficiency (couplant required). The coupling surface of the piezoelectric transducer is small in size [29]. The point or localized area of contact between the piezoelectric transducers and the cable surface makes it difficult to form a uniform excitation, which is not conducive to the generation of pure longitudinal mode-guided waves. The coupling efficiency may be reduced by dryness or failure of the couplant during long-term application. Guided wave generation relies on the contact between adjacent wires to transfer waves into inner layers from the outermost layer, where the transducers are installed [34]. The coupling efficiency of the piezoelectric transducers is mainly affected by the stability of the contact surfaces between the transducer and the wire bundle of the ACSR cable. Therefore, the field application of piezoelectric transducers in long-term SHM is limited by the possibility of slippage and abscission under wind loads. Although the piezoelectric polyvinylidene fluoride (PVDF) transducer [35] is flexible, the low energy-conversion efficiency limits the application in ACSR cable.

In addition to the above mentioned two types of transducers, electromagnetic acoustic transducer (EMAT) [36,37] and laser-based ultrasonic transducer [38,39] are also commonly used to excite and receive ultrasonic wave in industrial applications. The major advantage of EMAT is that they are non-contacting, coupling-free, and efficient to generate horizontal shear (SH)-guided wave [40], which is particularly important when testing high temperature structures [41]. However, EMAT gives relatively low transmitted ultrasonic energy, with low signal-to-noise ratio, and the induced energy is critically dependent on the transducer proximity to the test object. Well established applications of laser ultrasonic system are composite inspections for the aerospace industry and online high temperature pipe thickness measurements for the metallurgical industry [42]. However, the complexity and high cost of laser ultrasonic equipment has limited its application in ACSR cable online monitoring.

Due to the small saturation magnetostriction coefficient of the wires in cable, the application of long-distance structural monitoring using guided waves is limited. In recent years, researchers developed cobalt ferrite composites [43] with certain advantages, such as: large saturation magnetostrictive coefficient, high sensitivity, and high electrical resistivity. Plasma spraying has been used in making ferrite coatings and has been shown to be useful for magnetoresistance sensors [44]. Cold spraying has been used in making magnetostrictive commercially pure nickel cold spray patch sensor [45,46] for long-term crack monitoring of plates.

For a better performance of UGW inspection techniques on ACSR cables in engineering applications, an alternative sprayed magnetostrictive powder coating (SMPC) transducer is developed for guided wave generation and detection. The Fe_83_Ga_17_ alloy powder with large magnetostriction was sprayed uniformly on the surface of a section of cable where the transducer would be installed on site. Experimental investigations were carried out to generate and receive the fundamental longitudinal L(0,1) guided wave. The results demonstrate that the transducer with SMPC enhanced the amplitudes of the guided wave signals compared with the conventional transducer. The discernable reflected waves of the ACSR cable ends and the defects of three-wire breakage were received by the transducer with SMPC, which was impossible for the transducer without SMPC. This method makes long-term and online monitoring of ACSR cable feasible due to the high coupling efficiency and good structural surface adaptability.

## 2. Theory of Ultrasonic Guided Waves (UGW)

### 2.1. Aluminum Conductor Steel-Reinforced (ACSR) Cable Information

**Figure 1:**
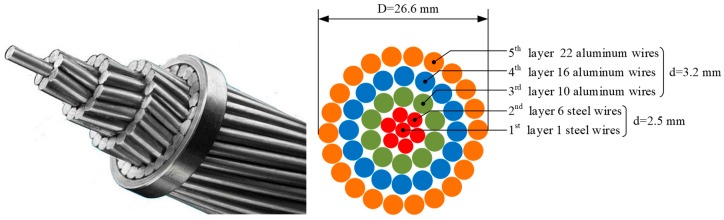
Photo and schematic diagram of LGJ-400/35 aluminum conductor steel-reinforced (ACSR) cable cross-section.

ACSR cables are widely used in power transmission line cables. They are composed of a number of twisted steel and aluminum wires. The diameter and number of individual aluminum and steel wires vary according to the model of the cable. The specimen tested in this experiment was a 1.6 meter-long LGJ-400/35 ACSR cable (Tianhong Electric Power Fitting Co., Ltd., Zhejiang, China). The cable is composed of 7 steel wires for load carrying and 48 aluminum wires for electric conduction, which are distributed as 5 layers: 1-6-10-16-22, from internal to external. The wire bundle is arranged into a helical shape with each layer twisted in the opposite direction. The diameters of the individual steel and aluminum wires are 2.5 mm and 3.2 mm, respectively. The overall diameter of the entire cable is 26.6 mm. The cross-section of the cable is shown in Figure 1.

### 2.2. Dispersion Curves of Guided Waves

UGWs travel with different velocities depending on the frequency of the wave. Group velocity dispersion curves are needed to determine the wave propagation speed for each mode. With this information, it is possible to convert the time-of-flight (ToF) into the distance traveled by wave packets in a structure. There are several techniques for calculating the dispersion curves of guided waves, such as the analytical method based on Pochhammer–Chree equations [47] and the semi-analytical finite element (SAFE) method [48]. The SAFE method is more attractive for analyzing a structure with an arbitrary geometry of the cross-section. The dispersion curves of multi-wire strands were obtained by Treyssède et al. They found that the group velocity curves of the fundamental order modes, such as L(0,1), T(0,1), and F(1,1), of the multi-wire helical structures with small helix lay angles (less than 15°) at low frequencies are very similar to the straight and helical wires [49,50,51]. The cable tested in this study is consistent with this situation.

The case of a single straight wire can be considered to be the same as a simple rod, whose dispersion properties can be analytically obtained using Pochhammer–Chree equations. The phase velocity and group velocity are derived from the (*ω*, *k*) solutions of the transcendental characteristic equation for longitudinal waves given by:

(1) $$\frac{2p}{a}\left(q^{2}+k^{2}\right)J_{0}\left(pa\right)J_{1}\left(qa\right)-\left(q^{2}-k^{2}\right)2J_{0}\left(pa\right)J_{1}\left(qa\right)-4k^{2}pqJ_{1}\left(pa\right)J_{0}\left(qa\right)=0$$

with,

$$p=\sqrt{\frac{\omega^{2}}{V_{L}^{2}}-k^{2}}\;\mathrm{and}\;q=\sqrt{\frac{\omega^{2}}{V_{S}^{2}}-k^{2}}$$

where *a*, *ω*, *k* are the rod radius, angular frequency, and wave number, respectively; *V_L_* and *V_S_* are the longitudinal and transverse velocities, respectively; and *J*_0_ and *J*_1_ are Bessel functions of order 0 and 1, respectively.

The phase and group velocity expressions are, respectively:

$$v_{ph}=\frac{\omega }{k}\;\mathrm{and}\;v_{g}=v_{ph}{\left(1-\frac{\omega }{v_{ph}}\frac{dv_{ph}}{d\omega }\right)}^{-1}$$

**Figure 2:**
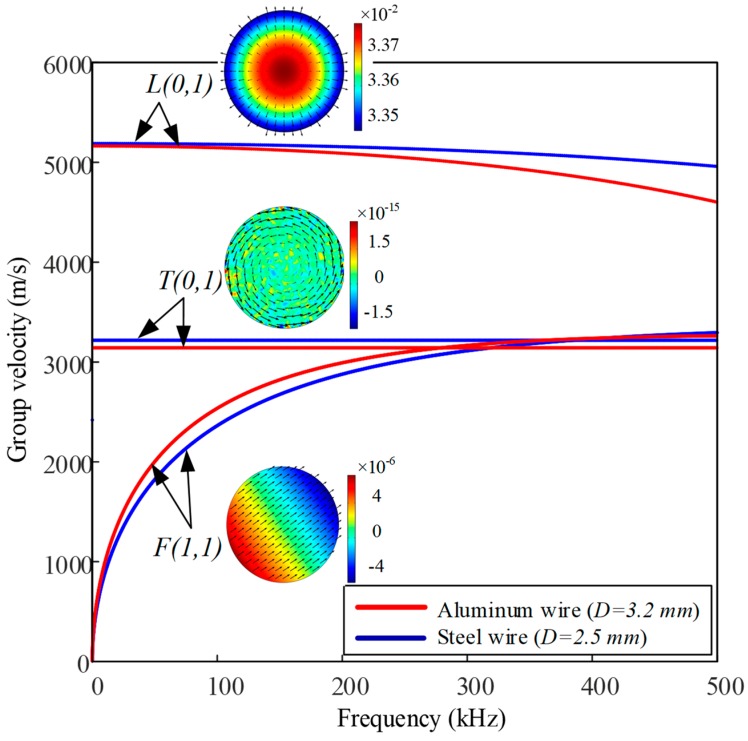
Group velocity dispersion curves of the lowest order longitudinal, torsional, and flexural wave modes for steel and aluminum wire at low frequency range.

The group velocity dispersion curves of individual steel wire with a diameter of 2.5 mm and aluminum wire with a diameter of 3.2 mm were acquired using a software package named PCDISP written in the Matlab (MathWorks Inc., Natick, NA, USA) environment, as shown in Figure 2. PCDISP is described in more detail in [52,53]. The material and geometric properties used in the dispersion models of PCDISP are summarized in Table 1.

Guided wave modes in a cylindrical waveguide are composed of longitudinal waves L(m,n), torsional waves T(m,n), and flexural waves F(m,n), where m ∈ {0, 1, 2, …} denotes the circumferential order and n ∈ {1, 2, 3, …} stands for the *n*th root of the characteristic equation [54]. Longitudinal wave mode has displacement in the radial and z-axial directions. Torsional wave mode has displacement in the circumferential direction. Flexural wave mode has displacement in all three directions [16]. All of the curves are dispersive in nature, although certain parts of the curves are flatter and less dispersive than the others, particularly in the low-frequency range for the low-order fundamental L(0,1) mode, which is the preferred mode for experiments and engineering applications. Another advantage of the longitudinal mode is the convenience of excitation and low-propagation attenuation. The group velocities of L(0,1) mode at frequencies of 50 kHz and 100 kHz are almost identical ($v_{g}=5065\;m/s$) with less dispersion.

## 3. Sprayed Magnetostrictive Powder Coating (SMPC) Transducer

### 3.1. Magnetostrictive Powder Spraying System

**Figure 3:**
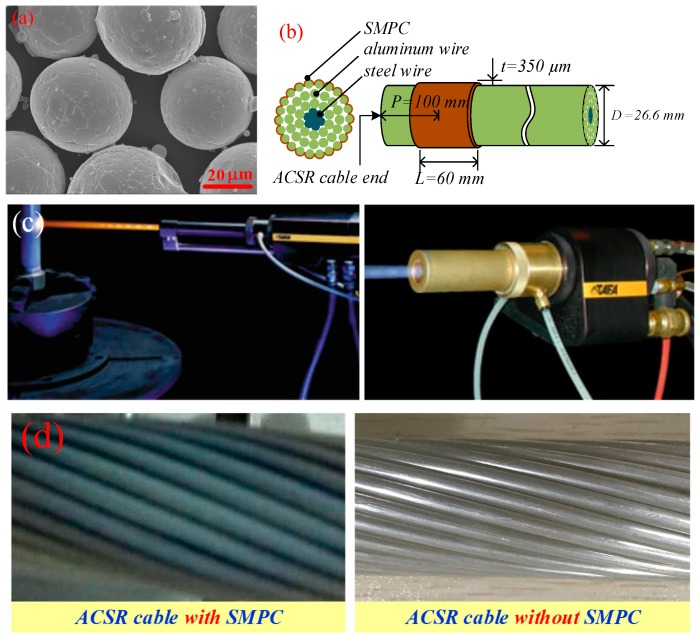
(**a**) Scanning electron microscope (SEM) micrograph of the Fe_83_Ga_17_ gas-atomized powder; (**b**) diagram of sprayed magnetostrictive powder coating (SMPC); (**c**) Photo of the spraying system; (**d**) aluminum conductor steel-reinforced (ACSR) cable with and without SMPC.

The feedstock of the coating was Fe_83_Ga_17_ gas-atomized powders with particle diameters between 30 and 50 µm, which were characterized by almost perfect spherical particles, as shown by the scanning electron microscope (SEM) photograph in Figure 3a. The coatings were sprayed by high-pressure, high-velocity oxygen fuel-spraying equipment (SX-JP8000, Siemens Co. Ltd., Munich, Germany). The torch was provided by a Praxair-TAFA 5220 (Praxair S.T. Technology, Inc., CT, Danbury, USA) with kerosene and oxygen as fuel gases, as shown in Figure 3c. Prior to spraying, the cable surface was cleaned using acetone solution and preheated to 100–200 °C, and then sandblasted with corundum powder. The cable was placed in a stainless steel cylindrical holder, and the axis of the torch was orthogonal to that of the holder with a rotating speed of 30 rpm. The spray distance was kept at 300 mm.

The coatings were uniformly deposited to a thickness of t = 350 µm on the outermost aluminum wire surfaces of the cable, as shown in Figure 3b. In order to compare the magnetostrictive energy conversion performance of the SMPC, only one cable end was sprayed, as shown in Figure 3d. The length of the SMPC (L = 60 mm) was slightly wider than the width of the UGW transducer coil used in the follow-up experiments. To facilitate transducer installation, the distance from the center of the spray area to the wire end was P = 100 mm.

**Figure 4:**
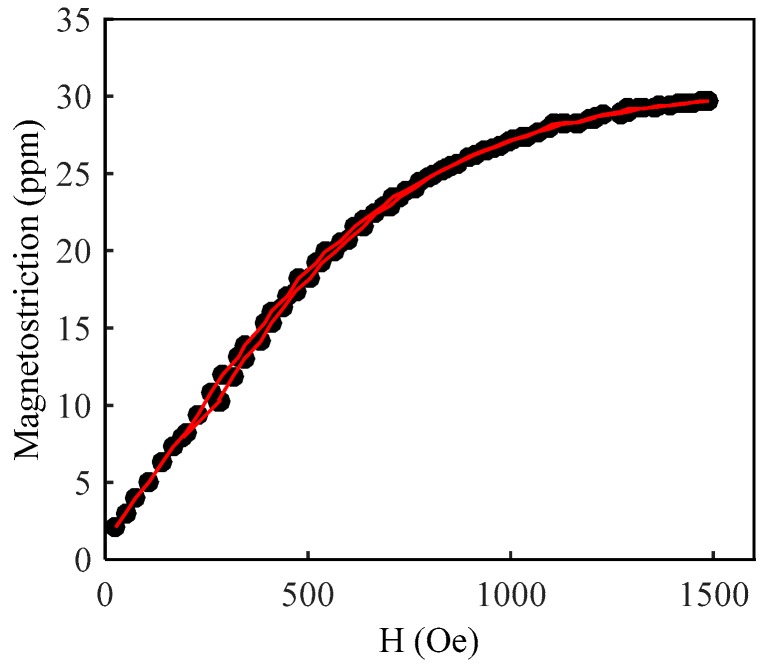
Magnetostriction versus applied magnetic field for Fe_83_Ga_17_ alloy powder coating.

In order to evaluate the magnetostriction of the SMPC, the same sprayed system and parameters were employed to spray a coating on an aluminum plate with a thickness of *t* = 350 µm. A resistance strain gauge positioned along the coating plane with a gauge area of 2.8 × 2.0 mm^2^ (base area of 6.4 × 3.5 mm^2^) was used to measure the magnetostriction. The magnetostriction of the as-deposited coating reached 30 ppm under a maximum external magnetic field of 1500 Oe at room temperature, as shown in Figure 4.

### 3.2. Working Mechanism

In a non-ferromagnetic conductive material subject to a static bias magnetic field, an applied dynamic magnetic field induces the Lorentz force [55] within it, thereby generating mechanical guided waves. In the case of a ferromagnetic material, an applied magnetic field induces magnetostriction [56] as well as the Lorentz force.

**Figure 5:**
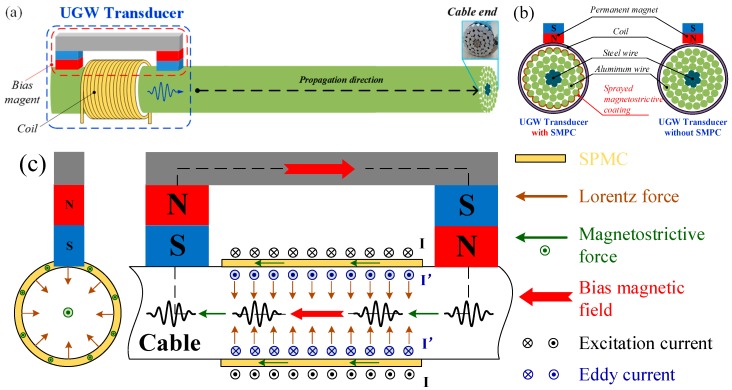
Generation of a longitudinal wave in ACSR cable. (**a**) Schematic of the transducer; (**b**) cross-sectional configuration of the transducer; (**c**) working mechanism.

When an ACSR cable without SMPC was subjected to a dynamic magnetic field (provided by an alternating current coil) superimposed on a static magnetic field (provided by a permanent bias magnet), the Lorentz force was generated in the outermost aluminum wires due to the eddy current effect. Longitudinal guided waves were excited by the Lorentz force in the direction along the radius of the cable. Axial strain was also produced in the innermost steel wires of the ACSR cable due to the magnetostrictive effect, but this became too weak to generate longitudinal guided waves. Bias magnetic field was attenuated by the three layers of aluminum wire, as shown in Figure 5.

As for an ACSR cable sprayed with magnetostrictive powder coating, guided waves were generated on account of the superposition of Lorentz force and magnetostrictive force, but magnetostriction was the dominant mechanism of transduction due to the greater resistivity and smaller eddy current effect of the powder coating. The direction of the Lorentz force was along the radius of the cable and the direction of the magnetostrictive force was along the axial of the cable, which happened to be consistent with the displacement distribution of the longitudinal mode guided wave, as shown in Figure 5c. This caused mechanical strain in the wires of the cable through the eddy effect and the magnetostrictive effect, known as the Joule effect. Conversely, for detection, the inverse magnetostrictive effect, known as the Villari effect, enabled guided waves to be detected through modifications in the magnetic induction.

## 4. Experimental Setup

In order to investigate the performance of the Fe_83_Ga_17_ coating transducer used in NDT and online SHM on the ACSR cable, magnetostrictive longitudinal mode guided wave generation and detection experiments were conducted to obtain the reflected wave signals of the cable ends and artificial wire breakage defect.

**Figure 6:**
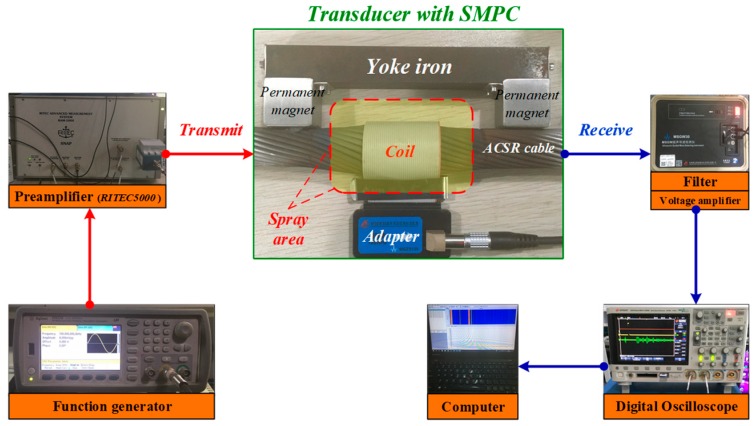
Experimental setup for ACSR cable detection using longitudinal magnetostrictive ultrasonic guided wave (UGW) transducer with SMPC.

The pulse-echo guided wave inspection configuration [57,58] was employed, as shown in Figure 6. Guided waves were generated in the experiments through a dynamic magnetic field (provided by a specially designed coil module) superimposed on a static magnetic field (provided by a permanent bias magnet). For versatility and ease of installation, the coil module consisted of a 50.8 mm wide 40-pin encircling ribbon cable and an adapter. The role of the adapter was to switch certain turns of the coil to be turned on to conveniently adjust the width of the wave source corresponding to the excitation frequency. The width of the conducting portion of the coil *W_en_* was equal to one-quarter wavelength λ of the guided wave at center excitation frequency, given by:

$$W_{en}=\frac{1}{4}\lambda =\frac{1}{4}\left(\frac{v_{g}}{f}\right)$$

where *v_g_* and *f* are group velocity and center excitation frequency, respectively.

The role of the bias magnet is to provide a static magnetic field that is consistent with the direction of the dynamic magnetic field and overcome the frequency-doubling effect of the ferromagnetic materials [32]. These two parallel magnetic fields will cause the wires in the cable to alternately expand and contract based on the magnetostrictive effect, thereby generating longitudinal guided waves. For bias magnet configuration, the components include two Ne–Fe–B N50 permanent magnets 30 × 40 × 30 mm^3^ in size and a yoke iron 30 × 120 × 25 mm^3^ in size.

**Figure 7:**
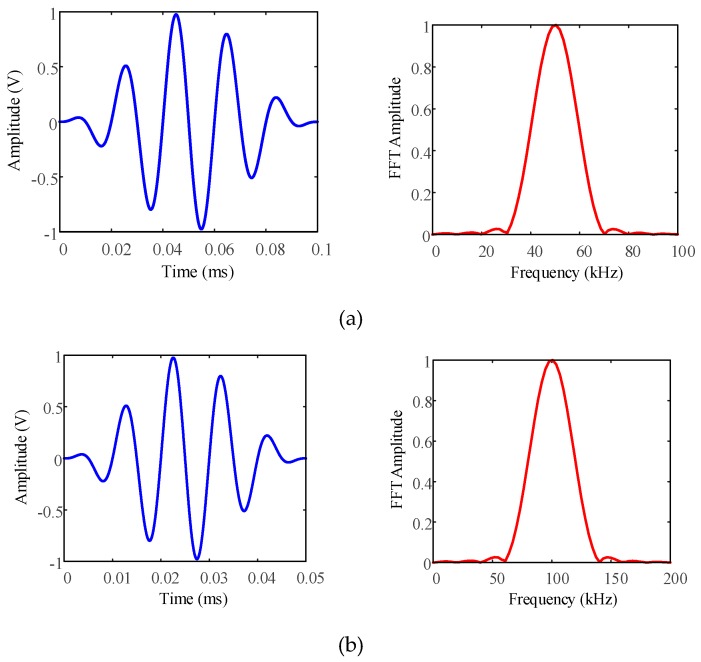
Excitation signal and fast Fourier transform (FFT) spectra of Hann-windowed 5-cycle (**a**) 50 kHz and (**b**) 100 kHz sinusoidal tone bursts.

The coil and the bias magnet were made up of the UGW transducer, which served as the transmitter and receiver simultaneously. These were used to generate and receive the L(0,1) guided waves at the center frequencies of 50 kHz and 100 kHz. For excitation, the coil was driven by a PC-controlled power amplifier (RAM-5000, Ritec Inc., Warwick, RI, USA) with a Hann-windowed 5-cycle sinusoidal tone burst, as shown in Figure 7. Meanwhile, the detected voltage signal in the coil was bandpass filtered and amplified by about 45 dB. The installation of the transducer with a distance of *L_T_* = 0.1 m close to one of the cable ends (with and without the SMPC) was carried out on the ACSR cable.

## 5. Results and Discussion

### 5.1. ACSR Cable End Detection

**Figure 8:**
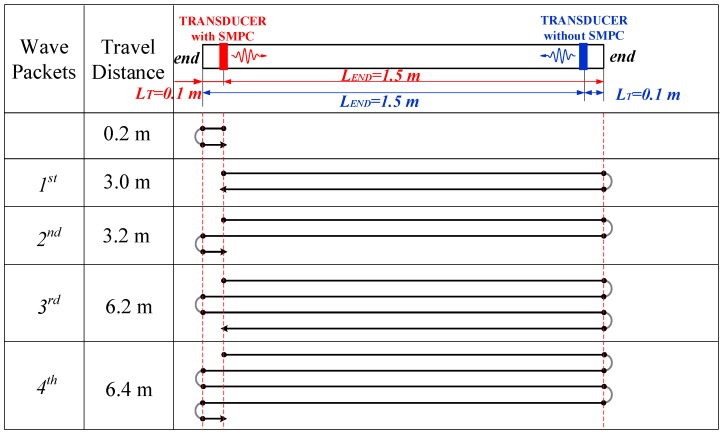
Schematic of pulse-echo detection experiments and propagation paths of waves excited by UGW transducer with SMPC.

Two sets of experiments were conducted on the 1.6 m long ACSR cable described in Section 2.1. The UGW transducers (with and without the SMPC) were placed respectively close to the two ends of the cable with a distance of *L_T_* = 0.1 m. The distance from the transducer to the far end was *L_END_* = 1.5 m. The propagation paths of guided waves associated with the configuration of transducers are shown in Figure 8. The figure shows all the propagation paths of the guided waves excited by the transducer with SMPC. They are similar to the transducer without SMPC, since the distances of the two transducers between the installation locations and cable ends were the same.

**Figure 9:**
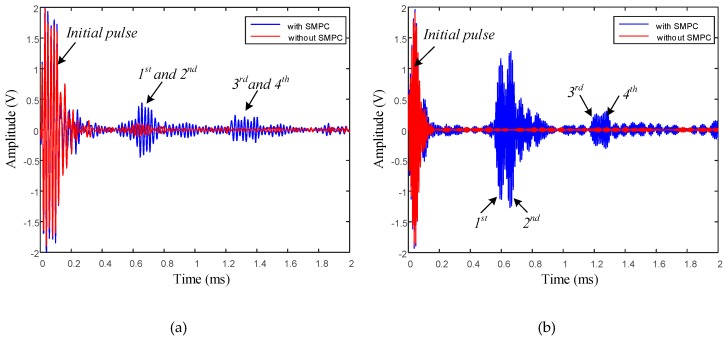
Signals obtained at transducer installed on ACSR cable with frequency of (**a**) 50 kHz and (**b**) 100 kHz.

The plots in Figure 9 show the time domain waveforms received by the transducers installed near two ends with center excitation frequencies of 50 kHz and 100 kHz. Signals of the end-reflected waves were not received by the transducer installed on the cable end without SMPC. This is because of the nonmagnetostriction of the aluminum wires and the inability of the magnetic field distribution of the bias magnet to reach into the steel wires located in the inner layers of the cable due to the liftoff distance. This also demonstrates that the guided wave excited by the eddy current effect was so weak that no guided wave could be generated in the cable. Conversely, the discernable reflected waves of the cable ends were received by the transducer installed on the cable end with SMPC.

The first portion of received signals consist of the initial electromagnetic pulse and superposition of the near-end reflection wave. The other four wave packets correspond to the multiple-end reflected waves with various acoustic travel paths, as summarized in Figure 8. The temporal resolution of guided wave detection increases as the excitation frequency increases, since the higher the frequency, the shorter the wavelength. All four wave packets can be clearly distinguished in the signals (transducer with SMPC at 100 kHz excitation frequency) in Figure 9b corresponding to the propagation paths of the guided waves. However, in Figure 9a, the first and second wave packets are difficult to distinguish, as are the third and fourth wave packets.

The amplitudes of the wave packets decrease with an increased wave travel distance. This is attributed to the acoustic energy attenuation caused by multiple total reflections of cable ends. The estimated end-reflected wave packet travel distances and amplitudes for a transducer with frequencies of 50 kHz and 100 kHz are summarized in Table 2. It is worth pointing out that the signal-to-noise ratio of the cable end-reflected wave after repeatedly passing through another end was reduced, caused by the contact and friction stresses between the adjacent wires in the cable.

Time of flight (ToF) represents the time history of the guided wave from excitation to reception as indicated by the abscissa of wave signals. The wave packet travel distances were calculated by Equation (2):

(2) $$d=t\left(f\right)\times v_{g}\left(f\right)$$

where *d* is the estimated distance, *t*(*f*) is the ToF of each wave packet at the frequency *f*, and $v_{g}\left(f\right)$ is the group velocity of L(0,1) mode at f = 50 kHz or 100 kHz.

None identifiable end-reflected wave packet was received by the transducer without SMPC, so the amplitudes are denoted by N/A (not available) in Table 2. The estimated distances of the wave packets were very close to the exact distances, with less than 5% error, which demonstrates the accuracy of the theoretical dispersion curves. The estimated acoustic distance of the cable end-reflected wave was slightly bigger than the exact distance due to the helical arrangement of the aluminum wires in the cable.

### 5.2. ACSR Cable Defect Detection

The UGW-based method is able to detect and monitor defects because reflections of waves occur when they encounter structural discontinuities. In multi-wire structures, structural discontinuities lead to changes in cross-sectional area (CSA), such as wire breakage.

**Figure 10:**
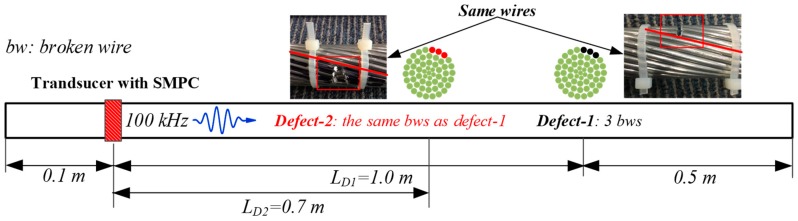
Schematic of pulse-echo detection experiment of cable defect using UGW transducer with SMPC.

In order to investigate the applicability of the UGW transducer with SMPC to detect defects in the ACSR cable, artificial defects were made on the cable used in the previous experiments. The experiments of defects detection were carried out in two stages, as shown in Figure 10. Each stage of broken wires is summarized as:

Stage 0: Same as the case in Section 5.1. (No defect)

Stage I: Stage 0 and Defect-1. Defect-1 was a saw cut of three wires (5.5% reduction in CSA), which was machined into the cable at a distance of L_D1_ = 1.0 m from the transducer.

Stage II: Stage I and Defect-2. Defect-2 was a saw cut of the same three wires as Defect-1, which was machined into the cable at a distance of L_D2_ = 0.7 m from the transducer.

**Figure 11:**
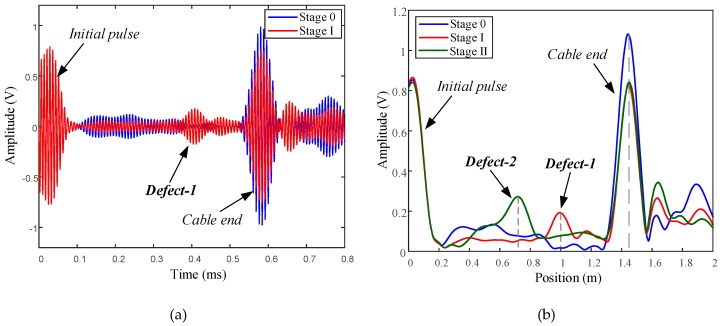
Reflected waves received by transducer installed on ACSR cable with artificial defect: (**a**) time domain signals; (**b**) Hilbert envelope signals.

The time domain signals received at the transducer with a center excitation frequency of 100 kHz is shown along with the Hilbert envelope in Figure 11. Three wave packets represent the reflected waves of two saw cut defects and cable end in sequence. At stage I, the reflected wave of Defect-1 was received. As the defect was introduced, the peak amplitude of the cable end wave packet decreased correspondingly, compared with that with no defect at stage 0. At stage II, after wave passes through Defect-2, the wave energy in these three wires was approximately equal to zero because total reflection occurs at saw cut. Therefore, only the reflected wave packet of Defect-2 was received in the signals. The peak amplitude of the cable end reflected wave packet was almost equal to that of Stage I, because the number of broken wires was same for two defects. The results are summarized in Table 3, which shows the practicability of defect detection and localization of the transducer with SMPC.

## 6. Conclusion

In this study, an alternative sprayed magnetostrictive powder-coated UGW transducer was developed for the inspection and online monitoring of overhead transmission line cables. The Fe_83_G_17_ magnetostrictive powder with particle diameters ranging from 30 to 50 µm was uniformly sprayed onto the surface of an ACSR cable with a thickness of 350 µm. A magnetostrictive longitudinal guided wave transducer was installed at the corresponding position of the spraying area of the cable. It could increase the detection range of UGW and amplitudes of the wave signals. The discernable reflected waves of the cable end and defect of a three-wire breakage were received by the transducer with SMPC, which was impossible for the transducer without SMPC. This makes long-term and online monitoring of the ACSR cables feasible due to the high coupling efficiency and good structural surface adaptability. Combining the SMPC and wire surfaces on a molecular level ensures stable coupling and energy conversion, which is the basic requirement of a UGW transducer applied in the NDT and SHM. SMPC transducers can also be used for SHM of other irregular structures with large curvature in which it is difficult to excite guided waves using conventional transducers, such as steel wire ropes, rails, heat exchange tubes, and so on.

During the thermal spraying process, high temperature may affect the mechanical properties of the aluminum wires of the ACSR cable. More work is needed to investigate the effects and other spraying parameters and methods, such as cold spraying.

## Figures and Tables

**Table 1:** Material and geometric properties used in PCDISP.

Material	Diameter	Density	Young Modulus	Poisson Ratio
Steel	2.5 mm	7800 kg/m^3^	209 GPa	0.3
Aluminum	3.2 mm	2700 kg/m^3^	72 GPa	0.35

**Table 2:** Estimated ACSR cable end-reflected wave packet travel distances and amplitudes for transducer with frequencies of 50 kHz and 100 kHz. ToF, time of flight. N/A, not available.

Transducer Frequency (kHz)	50 kHz				100 kHz			
Wave packet	1st	2nd	3rd	4th	1st	2nd	3rd	4th
Exact distance (m)	3.0	3.2	6.2	6.4	3.0	3.2	6.2	6.4
ToF (ms)	0.632		1.291		0.597	0.662	1.215	1.282
Group velocity (m/s)	5065		5065		5065	5065	5065	5065
Estimated distance (m)	3.201		6.539		3.024	3.353	6.154	6.493
Errors (%)	/		/		0.8%	4.8%	0.7%	1.5%
Without SMPC amplitude (V)	N/A		N/A		N/A	N/A	N/A	N/A
With SMPC amplitude (V)	0.442		0.449		1.161	1.28	0.261	0.302

**Table 3:** Estimated ACSR cable end and defects reflected wave packets travel distances for SMPC transducer with frequency of 100 kHz. CSA, cross-sectional area.

Experimental Stage	Stage 0	Stage I		Stage II	
Wave packet	Cable End	Defect-1	Cable End	Defect-2	Cable End
Exact position (m)	1.5	1.0	1.5	0.7	1.5
Exact distance (m)	3.0	2.0	3.0	1.4	3.0
ToF (ms)	0.573	0.391	0.573	0.279	0.573
Group velocity (m/s)	5065	5065	5065	5065	5065
Travel distance (m)	2.902	1.980	2.902	1.413	2.902
Estimated location (m)	1.451	0.990	1.451	0.707	1.451
Amplitude (V)	1.054	0.243	0.791↓	0.251	0.793↓

## References

[1] Yang L., Hao Y.P., Li W.-G., Li Z.-T. (2010). Relationships Among Transmission Line Icing, Conductor Temperature and Local Meteorology Using Grey Relational Analysis. High Volt. Eng..

[2] Paluch M., Cappellari T., Riera J. (2007). Experimental and numerical assessment of EPS wind action on long span transmission line conductors. J. Wind. Eng. Ind. Aerodyn..

[3] He J., Tu Y., Zeng R., Lee J., Chang S., Guan Z. (2005). Numeral Analysis Model for Shielding Failure of Transmission Line Under Lightning Stroke. IEEE Trans. Power Deliv..

[4] Zhao C., Chen J., Gu S., Ruan J., Li X. (2011). Analysis On the Lightning Trip-Out Failure of Transmission Line Under Conditions of Complex Landscape in a Mountainous Area. Power Syst. Technol..

[5] Chen J., Hu J., Xie Y., Li M., Liu F. (2013). Corrosion Failure of Fittings in Electric Power Transmission Line in Typical Industrial Areas. Corros. Sci. Protect. Technol..

[6] Wang J., Xiong X., Li Z., Liang Y., Weng S. (2016). Time Distribution of Weather-Related Transmission Line Failure and its Fitting. Electric Power Autom. Equip..

[7] Shull P.J. (2016). Nondestructive Evaluation: Theory, Techniques, and Applications.

[8] Zhang J., Guo Z., Jiao T., Wang M. (2018). Defect Detection of Aluminum Alloy Wheels in Radiography Images Using Adaptive Threshold and Morphological Reconstruction. Appl. Sci..

[9] Omidi P., Zafar M., Mozaffarzadeh M., Hariri A., Haung X., Orooji M., Nasiriavanaki M. (2018). A Novel Dictionary-Based Image Reconstruction for Photoacoustic Computed Tomography. Appl. Sci..

[10] Qin L., Ren H., Dong B., Xing F. (2015). Development of technique capable of identifying different corrosion stages in reinforced concrete. Appl. Acoust..

[11] Cao Y.-J., Wang J.-D., Liu W., Yang Y.-R. (2009). Wall sheeting diagnosis in fluidized beds based on chaos analysis of acoustic emission signals. J. Zhejiang Univ. Sci. A.

[12] Jian J., Xiao-Jun Z., Tian-Tai G., Si-Yuan W. (2005). Research on ultrasonic detection of complex surfaces. J. Zhejiang Univ. Sci. A.

[13] Shin E.-J., Kang B., Chang J.H. (2018). Real-Time HIFU Treatment Monitoring Using Pulse Inversion Ultrasonic Imaging. Appl. Sci..

[14] Zhang J., Tan X., Zheng P., Passaro V.M.N. (2017). Non-Destructive Detection of Wire Rope Discontinuities from Residual Magnetic Field Images Using the Hilbert-Huang Transform and Compressed Sensing. Sensors.

[15] Huang P.-j., Wu Z.-t. (2004). Inversion of Thicknesses of Multi-Layered Structures from Eddy Current Testing Measurements. J. Zhejiang Univ. Sci. A.

[16] Rose J.L. (2014). Ultrasonic Guided Waves in Solid Media.

[17] Yao Z.-J., Yu G.-L., Wang Y.-S., Shi Z.-F., Li J.-B. (2010). Propagation of flexural waves in phononic crystal thin plates with linear defects. J. Zhejiang Univ. Sci. A.

[18] Zhang X.-W., Tang Z.-F., Lv F.-Z., Pan X.-H. (2016). Excitation of axisymmetric and non-axisymmetric guided waves in elastic hollow cylinders by magnetostrictive transducers. J. Zhejiang Univ. Sci. A.

[19] Kwun H., Hanley J.J., Bartels K.A. Recent developments in nondestructive evaluation of steel strands and cables using magnetostrictive sensors. Proceedings of the OCEANS 96 MTS/IEEE Conference, The Coastal Ocean—Prospects for the 21st Century.

[20] Raisutis R., Kazys R., Mazeika L., Zukauskas E., Samaitis V., Jankauskas A. (2014). Ultrasonic guided wave-based testing technique for inspection of multi-wire rope structures. NDT E Int..

[21] Schaal C., Bischoff S., Gaul L. (2015). Energy-based models for guided ultrasonic wave propagation in multi-wire cables. Int. J. Solids Struct..

[22] Raisutis R., Kazys R., Mazeika L., Samaitis V., Zukauskas E. (2016). Propagation of Ultrasonic Guided Waves in Composite Multi-Wire Ropes. Materials.

[23] Treyssède F. (2016). Dispersion curve veering of longitudinal guided waves propagating inside prestressed seven-wire strands. J. Sound Vib..

[24] Gaul L., Bischoff S., Sprenger H., Haag T. (2010). Numerical and experimental investigation of wave propagation in rod-systems with cracks. Eng. Fract. Mech..

[25] Ke W., Peng X., Chen R., Chen H., Guo X. (2014). Unmanned Aerial Vehicle Platform Selection for Overhead Transmission Line Inspection. Electric. Power Sci. Eng..

[26] Peungsungwal S., Pungsiri B., Chamnongthai K., Okuda M. (2001). Autonomous Robot for a Power Transmission Line Inspection. IEEE Int. Symp. Circuits Syst..

[27] Hernandez-Salazar C.D., Baltazar A., Mijarez R., Solis L., Thompson D.O., Chimenti D.E. (2010). Structural Damage Monitoring On Overhead Transmission Lines Using Guided Waves and Signal Processing. Rev. Quant. Nondestruc. Eval..

[28] Baltazar A., Hernandez-Salazar C.D., Manzanares-Martinez B. (2010). Study of wave propagation in a multiwire cable to determine structural damage. NDT E Int..

[29] Legg M., Yücel M.K., Kappatos V., Selcuk C., Gan T.-H. (2015). Increased range of ultrasonic guided wave testing of overhead transmission line cables using dispersion compensation. Ultrasonics.

[30] Qiu J., Ji H., Wang E., Takagi T., Uchimoto T., Cheng J. (2016). High precision ultrasonic guided wave technique for inspection of power transmission line. Chin. J. Mech. Eng..

[31] Cho S.H., Lee J.S., Kim Y.Y. (2006). Guided wave transduction experiment using a circular magnetostrictive patch and a figure-of-eight coil in nonferromagnetic plates. Appl. Phys. Lett..

[32] Kim Y.Y., Kwon Y.E. (2015). Review of magnetostrictive patch transducers and applications in ultrasonic nondestructive testing of waveguides. Ultrasonics.

[33] Bartels K.A., Kwun H., Hanley J.J. (1996). Magnetostrictive sensors for the characterization of corrosion in rebars and prestressing strands. Proc. SPIE.

[34] Haag T., Beadle B.M., Sprenger H., Gaul L. (2009). Wave-based defect detection and interwire friction modeling for overhead transmission lines. Arch. Appl. Mech..

[35] Lin B., Giurgiutiu V. (2006). Modeling and testing of PZT and PVDF piezoelectric wafer active sensors. Smart Mater. Struct..

[36] Gao H., Ali S., López B. (2010). Efficient detection of delamination in multilayered structures using ultrasonic guided wave EMATs. NDT E Int..

[37] Wilcox P., Lowe M., Cawley P. (2005). Omnidirectional guided wave inspection of large metallic plate structures using an EMAT array. IEEE Trans. Ultrason. Ferroelectr. Freq. Control..

[38] Gao W., Glorieux C., Thoen J. (2003). Laser ultrasonic study of Lamb waves: Determination of the thickness and velocities of a thin plate. Int. J. Eng. Sci..

[39] An Y.-K., Park B., Sohn H. (2013). Complete noncontact laser ultrasonic imaging for automated crack visualization in a plate. Smart Mater. Struct..

[40] Seung H.M., Park C.I., Kim Y.Y. (2016). An omnidirectional shear-horizontal guided wave EMAT for a metallic plate. Ultrasonics.

[41] Hernandez-Valle F., Dixon S. (2010). Initial tests for designing a high temperature EMAT with pulsed electromagnet. NDT E Int..

[42] Monchalin J. (2004). Laser-Ultrasonics: From the Laboratory to Industry. AIP Conf. Proc..

[43] McCallum R.W. (2001). Composite magnetostrictive materials for advanced automotive magnetomechanical sensors. Low Temp. Phys..

[44] Gambino R., Raja M., Sampath S., Greenlaw R. (2004). Plasma-Sprayed Thick-Film Anisotropic Magnetoresistive (AMR) Sensors. IEEE Sens. J..

[45] Glass S.W., Lareau J.P., Ross K.S., Ali S., Hernandez F., Lopez B. Magnetostrictive Cold Spray Sensor for Harsh Environment and Long-Term Condition Monitoring. Proceedings of the 45th Annual Review of Progress in Quantitative Nondestructive Evaluation.

[46] Glass S.W., Lareau J.P., Ross K.A. (2018). Magnetostrictive cold spray coating for enhanced ultrasonic inspection. U.S. Patent.

[47] Gazis D.C. (1959). Three-Dimensional Investigation of the Propagation of Waves in Hollow Circular Cylinders. I. Analytical Foundation. J. Acoust. Soc. Am..

[48] Marzani A., Viola E., Bartoli I., Di Scalea F.L., Rizzo P. (2008). A semi-analytical finite element formulation for modeling stress wave propagation in axisymmetric damped waveguides. J. Sound Vib..

[49] Treyssède F., Laguerre L. (2010). Investigation of elastic modes propagating in multi-wire helical waveguides. J. Sound Vib..

[50] Laguerre L., Treyssède F. (2011). Non destructive evaluation of seven-wire strands using ultrasonic guided waves. Eur. J. Environ. Civ. Eng..

[51] Treyssède F. (2008). Elastic waves in helical waveguides. Wave Motion.

[52] Seco F., Martín J.M., Jiménez A., Pons J.L., Calderón L., Ceres R. Pcdisp: A Tool for the Simulation of Wave Propagation in Cylindrical Waveguides. Proceedings of the 9th International Congress on Sound and Vibration.

[53] Seco F., Jiménez A.R. (2012). Modelling the Generation and Propagation of Ultrasonic Signals in Cylindrical Waveguides. Ultrasonic Waves.

[54] Ditri J.J., Rose J.L. (1992). Excitation of guided elastic wave modes in hollow cylinders by applied surface tractions. J. Appl. Phys..

[55] Hirao M., Ogi H. (2013). Emats for Science and Industry: Noncontacting Ultrasonic Measurements.

[56] Lee E.W. (1955). Magnetostriction and Magnetomechanical Effects. Rep. Prog. Phys..

[57] Laguerre L., Aime J.-C., Brissaud M. (2002). Magnetostrictive pulse-echo device for non-destructive evaluation of cylindrical steel materials using longitudinal guided waves. Ultrasonics.

[58] Zhang P., Tang Z., Duan Y., Yun C.B., Lv F. (2018). Ultrasonic Guided Wave Approach Incorporating Safe for Detecting Wire Breakage in Bridge Cable. Smart Struct. Syst..

